# Gourmet Table Salts: The Mineral Composition Showdown

**DOI:** 10.3390/toxics11080705

**Published:** 2023-08-15

**Authors:** Eleonora Di Salvo, Roberta Tardugno, Vincenzo Nava, Clara Naccari, Antonio Virga, Andrea Salvo, Filomena Corbo, Maria Lisa Clodoveo, Nicola Cicero

**Affiliations:** 1 Departement of Biomedical and Dental Sciences and Morphofunctional Imaging, University of Messina, 98168 Messina, Italy; eleonora.disalvo@unime.it; 2 Department of Pharmacy-Drug Sciences, University of Bari, 70121 Bari, Italy; roberta.tardugno@uniba.it (R.T.);; 3 Department of Health Sciences, University “Magna Græcia” of Catanzaro, 88100 Catanzaro, Italy; c.naccari@unicz.it; 4 Department of Agricultural, Food and Forestry Sciences, University of Palermo, 90121 Palermo, Italy; antoninovirga58@gmail.com; 5 Department of Chemistry and Drug Technology, University of Roma La Sapienza, 00185 Roma, Italy; asalvo@uniroma1.it; 6 Science4life srl, University of Messina, 98168 Messina, Italy

**Keywords:** gourmet table salt, NaCl, mineral element, nutraceuticals, ICP-MS, TDA-AAS

## Abstract

Table salts with their specialty flake size, textures, flavors, and colors can be considered a gastronomy niche food already increasing in demand worldwide. Being unrefined, they can contain trace elements potentially both healthy and toxic. In this study, 12 mineral elements (Al, Ca, Co, Cr, Cu, Fe, Hg, Mn, Ni, Pb, Se, and Zn) in 10 different salts commercially available in southern Italy namely, Atlantic grey, Baule volante, Guerande, Hawaiian pink, Hawaiian black, Himalayan pink, Maldon, Mozia, Persian blue, and smoked salts were analyzed by inductively coupled plasma mass spectrometry (ICP-MS) and thermal decomposition amalgamation-atomic absorption spectrophotometry (TDA-AAS). The concentration of mineral elements was variable according to the type of salt and its geographical origin. Co, Cr, Cu, Hg, Pb, and Se levels were tolerable and Al, Ca, Fe, Mn, Ni, and Zn ranged significantly among the samples. Persian Blue and Atlantic Grey salts showed elevated levels of Fe and Zn; their intake can be helpful in some specific conditions. Nevertheless, Ni levels were high in Persian Blue and Smoked salts. Pb exceeded the maximum level in all samples. Additional monitoring analyses of mineral contents in table salts are recommended for human health.

## 1. Introduction

Sodium chloride (NaCl) commonly known as “table salt”, “kitchen salt”, is a type of refined salt used worldwide for culinary purposes. Table salt has been used as a food flavoring and as a preservative agent from ancient times. Salt is obtained through the evaporation of water from brine solution by a boiling process of seawater, salt spring water, or from salt rocks mining [1,2,3]. In general, salt contains sodium chlorine by weight as the main salt element, an essential constituent for the osmotic balance of cells. In addition, salt is an excellent carrier of iodine (I) for the prevention of iodine deficiency disorders [4]. Table salt also contains many other essential minerals that are required for various physiological and metabolic processes in living organisms [1,2,3] such as calcium (Ca), cobalt (Co), copper (Cu), iron (Fe), magnesium (Mg), potassium (K), selenium (Se), zinc (Zn), and many others which may vary depending on the region and method of harvest [5,6].

The World Health Organization (WHO) has recommended a reduction in salt intake to 5 g of NaCl (or 2 g of Na) per day [4]. Consumer demands for “natural” products are increasing as well as the demand for the use of “gourmet” or “speciality” salts [7]. Namely, table salts with different colors (black, blue, grey, pink, etc.) and shapes (flakes, pyramidal, etc.) and gourmet salts have grown in popularity and are increasing in demand by home cookers and chefs globally due to their unique characteristics [1]. Some gourmet table salts can contain higher amounts of other mineral elements than the common table salt. Indeed, the higher presence of trace elements in certain gourmet table salts confers distinctive physical–chemical properties to each salt [8]. Indeed, the minor components of salts vary significantly according to the manufacturing process, the raw materials’ composition, and the collection site [1,2,3].

Being less refined, gourmet table salts alongside the presence of health-beneficial mineral elements can contain also potentially toxic elements such as heavy metals [7,9]. The presence of health-promoting trace minerals (Ca, Fe, K, Mg, and Zn) is important in biological pathways, cells, and consequently in certain physiopathological human conditions [10]. On the other hand, due to the daily consumption of table salt, any contamination in salt even at a low level could create health risks to the consumers [11,12]. The main reason for the accumulation in gourmet table salts of potentially toxic and toxic mineral elements including aluminum (Al), chromium (Cr), mercury (Hg), lead (Pb), Nickel (Ni), and others can be associated with the contamination of collection sites [2,9,11,13,14,15].

Recently, the incidence of heavy metal contamination in table salt was investigated worldwide; more monitoring is needed. Given the increasing rate of consumption of gourmet table salts, it is, therefore, appropriate to investigate both their nutritional value and level of toxicity [4,16].

Thus, in this context, the mineral content variability of gourmet table salts due to their potential nutritional, environmental, and economic value as a niche food plays a key role.

According to the Codex Alimentarius Commission, flame atomic absorption spectrophotometry (FAAS), inductively coupled plasma optical emission spectroscopy (ICP-OES), and inductively coupled plasma mass spectrometry (ICP-MS) are the most used techniques for trace element determination [4,17].

The objective of this study was to investigate the content of 12 mineral elements (Al, Ca, Co, Cr, Cu, Fe, Hg, Mn, Ni, Pb, Se, and Zn) with potentially beneficial and toxic properties in gourmet table salts commercially available in southern Italy markets in alphabetical order: Atlantic grey salt, Baule Volante Sicilian organic rock salt, Guerande French salt, Hawaiian black salt, Hawaiian pink salt, Himalayan pink salt, Maldon British salt, Mozia Sicilian sea salt, Persian blue salt, and Smoked salts. The chemical trace element profile of salt was analyzed with ICP-MS and TDA-AAS. As far as we know, this is the first time that these 10 salts, from such different regions, have been studied together to speculate their potential as nutraceuticals or toxins.

## 2. Materials and Methods

### 2.1. Salt Samples

In this study, there were 10 salt samples; each salt was analyzed in triplicate and those that were commercially available were purchased from different local markets in Messina (southern Italy) in May 2022.

**Figure 1:**
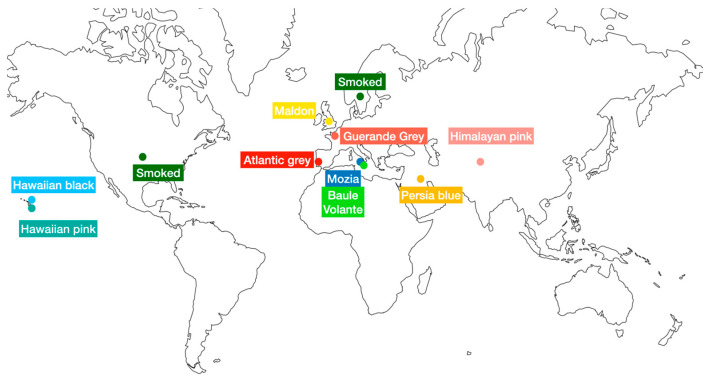
Salt samples’ origin area map.

The salts, namely Mozia, Atlantic grey sea, Persian blue, Smoked, Guerande Grey, Hawaii pink, Hawaii black, Himalayan pink, Maldon, and Baule Volante whole rock, were analyzed. Table 1 and Figure 1 show the samples investigated, referring to different types and their area of origin when indicated.

### 2.2. Materials and Reagents

Ultrapure water was purchased from Merck-Millipore (Darmstadt, Germany). Nitric acid (HNO_3_, 65% *v*/*v*) was purchased from J. T. Baker (Mallinckrodt Baker, Milan, Italy). From Supelco (Bellefonte, PA, USA), the commercial standard solution of Re (internal standard) and the standards of Al, Ca, Co, Cr, Cu, Fe, Mn, Ni, Pb, Se, and Zn, used for the calibration curves came, were purchased. From Merck (Darmstadt, Germany), a Hg solution (1000 mg/L in 3% hydrochloric acid) was purchased. To clean the DMA-80, a 3% HCl solution prepared from concentrated HCl (37%) and obtained from Merck (Darmstadt, Germany) was used.

### 2.3. Sample Preparation

In total, 1 kg of each salt was purchased from various local markets in Messina. The salts were sampled in plastic bags and then ground with a mortar and sieved to obtain homogeneous particle sizes.

For each sample, 5 g of salt was dissolved in 100 mL of ultrapure water and 1 mL of nitric acid (HNO_3_, 65% *v*/*v*) and left to rest for 3 days. To minimize the problem of selenium volatility, sample preparation was performed in closed vessels. Then, all the samples were filtered through 0.45 μm PTFE filters.

### 2.4. ICP-MS Analysis

Al, Ca, Co, Cr, Cu, Fe, Mn, Ni, Pb, Se, and Zn content was determined by the single quadrupole inductively coupled plasma-mass spectrometer (ICP-MS, iCAP-Q, Thermo Scientific, Waltham, MA, USA) powered by a 27 MHz radiofrequency solid-state generator and equipped with a PFA cyclonic spray chamber with a port accepting a 4 mm i.d. and 6 mm o.d. nebulizer, nickel sampler, and skimmer cones of 1.1 mm and 0.5 mm. The instrument was also provided with an autosampler (ASX520, Cetac Technologies Inc., Omaha, NE, USA) coupled with an integrated sample introduction system.

The following isotopes were monitored for ICP-MS analysis: ^27^Al, ^44^Ca, ^59^Co, ^52^Cr, ^63^Cu, ^56^Fe,^55^Mn, ^60^Ni, ^208^Pb, ^80^Se, and ^66^Zn.

Salt samples were analyzed under the following operating conditions: RF power, 1550 W; plasma gas (Ar) flow rate, 14 L/min; auxiliary gas (Ar), flow rate, 0.8 L/min; carrier gas (Ar) flow rate, 1.1 L/min; collision gas (He) flow rate, 4.7 mL/min; spray chamber temperature, 2.7 °C; and sample depth and sample introduction flow rate, 5 mm 0.93 mL/min.

The integration times were 0.5 s/point for Fe and Se and 0.1 s/point for the other elements.

Data acquisition was possible using the Thermo Scientific QtegraTM Intelligent Scientific Data System software (Thermo Scientific, Waltham, MA, USA). In addition, a seven-point calibration plot with an internal standard normalization (1 mL of internal Re standard at 0.5 mg L^−1^) was constructed for quantitative analysis. All samples were analyzed in triplicate.

### 2.5. DMA-80 Analysis

For salt samples, a determination of Hg content was also carried out with a direct mercury analyzer (DMA-80, Milestone S.r.l., Sorisole, Italy), an instrument based on the thermal decomposition amalgamation-atomic absorption spectrophotometry (TDA-AAS). The DMA-80 is a more versatile analytical instrumentation compared to ICP-MS: it permits the direct analysis of the sample without the need for pre-treatment and it is a more environmentally friendly analytical method than ICP-MS due to the use of a minimal amount of reagent for instrument cleaning and the safety of operators who are not exposed to mercury. The guidance reported by the EPA Method 7473 (SW-846) [18] was referred to in developing the method of analysis of DMA-80. Precisely, ~100 mg of every homogenized sample was initially dried at 230 °C for 3 min and then thermally decomposed at 650 °C for 3 min. The Hg content was determined by working at its typical wavelength, i.e., 253.7.

### 2.6. ICP-MS and the DMA-80 Validation Method

Method validation was carried out following Eurachem criteria [18], i.e., determining linearity, the limit of detection (LOD), the limit of quantification (LOQ), and accuracy (Table 2).

### 2.7. Statistical Analysis

We analyzed 10 different types of salts by dosing the levels of 12 diverse elements. Due to their quantity and the heterogeneity of their composition, the most suitable approach was adopting descriptive statistics (Table 3 and Table 4). We used a laptop Lenovo 330s-15-ARR with a Ryzen 5 processor and 20 GB of RAM. Our analysis was conducted by using IBM SPSS and Microsoft Excel 2022—version 16.66.1.

## 3. Results

In this study, 12 mineral elements (Al, Ca, Co, Cr, Cu, Fe, Hg, Mn, Ni, Pb, Se, and Zn) in gourmet table salts commercially available in southern Italy were analyzed by inductively coupled plasma mass spectrometry (ICP-MS) and thermal decomposition amalgamation-atomic absorption spectrophotometry (TDA-AAS).

The linearity was optimal over the concentration range tested with R^2^ > for all elements, as shown in Table 2. The reported LODs ranged from 0.001 to 1.211 µg/Kg while the LOQs ranged from 0.003 to 4.462 µg/Kg (Table 2), indicating good sensitivity of the method for sample analysis (Table 2). The accuracy of the analytical procedure, as assessed by the recovery test, was very good with percentage recoveries ranging from 91.36 to 101.50%. The accuracy of the method was evaluated by addition and recovery tests using two concentration levels. The calculation used to determine the percentage recovery was as follows:

R = 100(A2 − A1)/As

where R = recovery percentage; A1 = concentration of the sample without addition of standard; A2 = sample with the addition of standard; and As = concentration of the standard solution.

The lowest and the highest mean recoveries were observed for Ca (91.36 ± 2.33%) and Pb (101.50 ± 1.12%) (Table 2).

Quantitative data of the recovered mineral contents are presented in Table 3. It shows the mineral contents present in the analyzed salts. The mineral elements are listed in alphabetical order in Table 3 and all values are expressed in mg/Kg.

Aluminum (Al) contents were variable, ranging from 0.25 ± 0.05 mg/Kg (Persian blue salt) to 14.11 ± 1.03 mg/Kg (Mozia salt).

Calcium (Ca) was highly present in all samples. Its concentration varied from 1861.41 ± 3.98 mg/Kg in Hawaiian black salt to 6252.38 ± 4.20 mg/Kg in Persian blue salt.

Cobalt (Co) concentrations were determined for all samples and ranged from 0.20 ± 0.02 mg/Kg for Himalayan pink salt to 0.82 ± 0.07 mg/Kg for Guerande grey salt.

Persian blue salt had the highest chromium (Cr) content (1.53 ± 0.08 mg/Kg) while Maldon salt had the lowest (0.21 ± 0.01 mg/Kg).

Copper (Cu) was present in similar concentrations in almost all samples except for Mozia and Persian blue salts: 41.63 ± 4.18 mg/Kg and 50.61 ± 6.19 mg/Kg, respectively.

Iron (Fe) contents were very variable, ranging from 1.44 ± 0.26 mg/Kg in Hawaiian pink salt to 21.72 ± 2.11 mg/Kg in Persian blue salt.

For mercury (Hg), very low concentrations were obtained for all the salt samples analyzed, namely approximately 0.001 ± 0.000 mg/Kg for Atlantic grey sea, Hawaiian black, Maldon, Mozia, Persian blue and smoked salts and 0.04 ± 0.00 mg/Kg for Baule Volante salt.

Guerande grey salt had the highest manganese (Mn) content with 5.15 ± 0.62 mg/Kg while the Maldon salt had the lowest with 0.82 ± 0.04 mg/Kg.

Nickel (Ni) concentrations were very variable in the different samples, ranging from 0.21 ± 0.02 mg/Kg in Atlantic grey sea salt to 12.41 ± 1.78 mg/Kg in Persian blue salt.

Lead (Pb) levels were very high in all samples. Smoked salt had the lowest content (5.24 ± 0.28 mg/Kg) while Persian blue salt had the highest (9.24 ± 0.42 mg/Kg).

All samples contained similar amounts of selenium (Se), ranging between 0.20 ± 0.02 mg/Kg and 0.22 ± 0.02 mg/Kg.

High concentrations of zinc (Zn) were found in all samples. The values ranged from 156.38 ± 1.57 mg/Kg (Hawaiian black salt) to 1334.44 ± 8.17 mg/Kg (Persian blue salt).

The different geographical origins of the samples certainly influenced their mineral content (Table 5). This variability in mineral content could indeed be due to the profile and quality of the soil and rock in which the salt was harvested [19].

Of the salt types analyzed, Persian blue salt was the one with the highest mineral content. In fact, it had the highest levels of 8 of the 12 elements analyzed: Al, Ca, Cr, Cu, Fe, Ni, Pb, and Zn. Guerande grey salt had the highest concentrations of Co and Mn while Baule Volante salt, Mozia salt, and Hawaiian black salt had the highest levels of Hg, Se and Al, respectively.

## 4. Discussion

**Figure 2:**
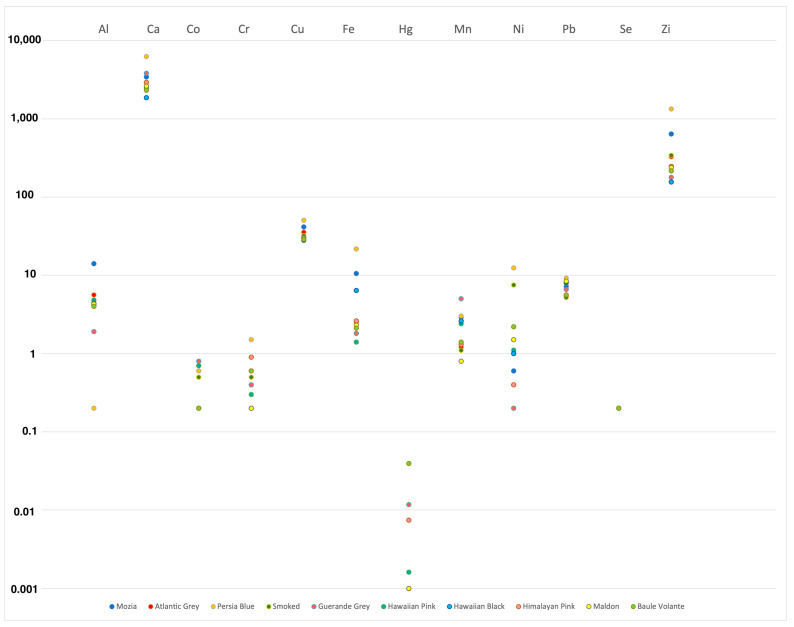
Elements concentration distribution expressed as Log^10^ in gourmet table salt samples analyzed from different geographic origins.

The concentration of mineral elements varied according to the type of salt and its geographical origin, as visually reported in Figure 2.

Significant levels of aluminum were found in almost all the salts analyzed. Many of the results obtained are comparable to those of the study by Lugendo et al. [20] on the determination of heavy metals and essential elements in table salts extracted from the Bahi wetlands in central Tanzania. In this study, the Al content ranged from 4441.00 ± 0.03 mg/Kg to 6484.00 ± 0.03 mg/Kg. Comparable results to our study were obtained by comparison with the study conducted by Abdi et al. [21] on the determination of trace metal contaminants in edible salts in Tehran (Iran) using atomic absorption spectrophotometry, in which the authors determined an average aluminum content between 5.60 ± 0.75 mg/Kg and 5.82 ± 0.61 mg/Kg. The different availability of Al in edible salts correlates with the different soils and rocks from which the different salts are extracted.

The macro element calcium shows a high level in each edible salt sample. All samples showed a lower Ca content (1861.41 ± 3.98–3818.14 ± 4.98 mg/Kg) than that reported by Lugendo et al. [20] (4443–5454 mg/Kg) except for the Persian blue salt (6252.38 ± 4.20 mg/Kg). However, when compared to Rehan et al. [22] on the determination of toxic and essential metals in rock and sea salts using pulsed nanosecond laser-induced breakdown spectroscopy and to Sani et al. [23] on the determination of radioactivity and elemental concentrations of natural and commercial salts, our average calcium levels were significantly higher (3129.59 mg/Kg vs. 233.33 mg/Kg and 88.99 mg/Kg).

The concentration of cobalt was less than 1 mg/Kg in all samples. These results were significantly lower than those reported by Lugendo et al. [20] (7.28–7.56 mg/Kg) and by Rehan et al. [22] (10–17 mg/Kg in edible salts) but much higher than those found in the study conducted by Eftekhari et al. [24] on the determination of toxic and essential metal contents in recrystallized and washed table salts in Shiraz, Iran (0.01 mg/Kg in recrystallized salts, while 0.04 mg/Kg in washed salts), and by Siulapwa et al. [25] on the evaluation of essential elements and trace metal contaminants in commercial and traditional table salts in Zambia (0.04–0.15 mg/Kg). The study by Nafees et al. [26] on the analysis of rock and sea salts for various essential and inorganic elements showed that two of the seven types of salt analyzed had cobalt contents of 10 mg/Kg significantly higher than those determined by us while the other was below the limit of quantification.

The chromium content varied according to the type of salt analyzed. Compared with the study by Sani et al. [23], the average Cr content was significantly lower: 0.58 mg/Kg vs. 11.75 mg/Kg. Similarly, Shariatifar et al. [27], in this study on the evaluation of heavy metal content in refined and unrefined salts obtained from Urmia (Iran), obtained a higher mean Cr concentration for unrefined salt than our results (2.46 mg/kg) but lower refined salt (0.33 mg/Kg). An opposite trend is observed when compared with Fayet-Moore et al. [1] who analyzed the mineral composition of pink salt available in Australia in which chromium was present at lower concentrations.

Copper is present in very high concentrations. Again, the geographical origin of the product is important. In fact, in the literature, there are different concentrations of Cu in salt samples depending on the origin of the matrix. For example, our results were higher than those of Khaniki et al. [28] (33.59 mg/Kg vs. 1.21–1.24 mg/Kg), Eftekhari et al. [24] (33.59 mg/Kg vs. 0.11–0.20 mg/Kg), Fayet-Moore et al. [1] (33.59 mg/Kg vs. 0.02–0.41 mg/Kg), Siulapwa et al. [25] (33.591 mg/Kg vs. 0.15–2.32 mg/Kg), and Nafees et al. [26] (33.59 mg/Kg vs. 4.04 mg/Kg). These significant concentrations are problematic. In fact, the Italian Ministry of Health decree [29] on the production and marketing of table salt stipulates that the copper content of this matrix must not exceed 2 mg/Kg. As a result of this legislation, all the samples analyzed were well above this legal limit.

Iron was always present in concentrations above 1 mg/Kg. The presence of this element is also closely linked to its availability in the environment. In fact, several studies in the literature report different concentration ranges for Fe. For example, Lugendo et al. [20], Sani et al. [23], Fayet-Moore et al. [1], and Rehan et al. [22] all showed higher concentrations: 57.21–709.50 mg/Kg, 43.9–132.5 mg/Kg, n.d.–167.52 mg/Kg, and 22–40 mg/Kg, respectively.

The new repealing regulation of the European Union [30] on maximum levels of certain contaminants in food regulation [31] sets maximum levels for certain inorganic elements in salt including mercury and lead. For Hg, the maximum level is 0.10 mg/Kg. Consequently, all the salt samples analyzed were well within this limit. For Pb, on the other hand, two different maximum levels are indicated depending on the class of salts: for salts in general, the maximum permitted level is 1.0 mg/Kg while for unrefined salts such as “fior di sale” and “grey salt”, the regulation sets a limit of 2.0 mg/Kg. In any case, our samples always exceeded the maximum permitted levels. This is not a good result considering that lead is a toxic element that accumulates in the body and affects different systems and organs such as the central and peripheral nervous system and the gastrointestinal tract [32]. The levels of mercury were low and comparable to those found by Fayet-Moore et al. [1] and by Abdi et al. [21] in their preliminary study of the concentration of potentially toxic elements (PTEs) in salt samples collected in Tehran (Iran) to assess their health risk. However, for lead, different studies show different concentrations of this element in different salts. This could be due to differences in the geographical origin of the product. For example, our salts were characterized by significantly higher lead levels than those reported by Eftekhari et al. [24] (0.31–0.38 mg/Kg), Fayet-Moore et al. [1] (<LOQ-2.59 mg/Kg), Mostafaii et al. [13] (concerning concentration determination and the risk assessment of potentially toxic elements (PTE) in unrefined salt from Lake Aran in Iran (0.15 mg/Kg)), and by Siulapwa et al. [25] (0.10 mg/Kg) but lower than those reported in the study by Abdi et al. [21] for refined salt (13.38 mg/Kg).

The manganese content ranged from 0.82 ± 0.04 to 5.15 ± 0.62 mg/Kg. These values were significantly lower than the study of Lugendo et al. [20] (29.13–85.60 mg/Kg), Sani et al. [23] (10.77 mg/Kg), and Rehan et al. [22] (15.0–22.0 mg/Kg), higher than those in the study of Shariatifar et al. [27] (0.18–0.19 mg/Kg), and comparable to those of Fayet-Moore et al. [1] (0.33–8.61 mg/Kg). This variability in Mn content is closely related to the soil type. In fact, areas with acid pH can contain a high percentage of Mn^2+^ ions [16].

Nickel content was also quite variable in relation to the type of salt analyzed. This is because this element is easily found in the soil, with concentrations ranging from 5 to 500 mg/Kg. Our results were significantly higher than most of the samples analyzed in the study conducted by Fayet-Moore [1] (0.01–0.42 mg/Kg) and in the study by Mostafaii et al. [13] (0.22 mg/Kg) while they were comparable to the work by Sani et al. [23] (2.1 mg/Kg).

Selenium was present in almost identical concentrations in all samples analyzed. There are not many references in the literature dealing with the determination of selenium content in table salt. However, in the Fayet-Moore et al. [1] study, this element was determined and the levels were lower (n.d.–0.19 mg/Kg) than in our study.

Finally, high concentrations of zinc were found in all salt samples. These were significantly higher than those reported in other studies: Khaniki et al. [28] (6.02–6.50 mg/Kg), Eftekhari et al. [24] (0.34–0.38 mg/Kg), Fayet-Moore et al. [1] (n.d.–0.86 mg/Kg), Sani et al. [24] (37.91 mg/Kg), and Lugendo et al. [20] (3.11–7.85 mg/Kg).

### 4.1. Geochemical Imprint in Salt Differences

The observed differences in trace element composition across a spectrum of commercial salts are influenced by a multitude of complex factors. The geographical origin, as indicated by the distinctive characteristics of salts such as Persian Blue, Guerande Grey, Maldon, and Atlantic Grey, suggests that regional geology, specifically the mineralogical attributes of the source location, significantly contributes to these variations. For instance, Persian Blue salt, sourced from Iranian salt mines, displays a high calcium and iron content likely reflecting the unique calcium and iron-rich geological formations inherent to this region. Similarly, the high copper content in Guerande Grey salt, Hawaiian Pink salt, and Smoked salt may be influenced by the presence of copper-enriched clay deposits or copper-rich seawater. Processing techniques also play a crucial role. The treatment involved in creating Smoked salt, which involves smoking over wood fires, may introduce or modify existing elements, thus diversifying its composition. The ‘purity’ factor as seen in Himalayan Pink salt, heralded as one of the purest due to its ancient origin and deep underground harvesting, may lead to lower levels of contemporary pollutants such as lead. In essence, each salt’s unique mineralogical profile is a testament to its geospatial origin, extraction, and processing narrative, underscoring the importance of these factors in nutritional and toxicological evaluations.

### 4.2. RDA: Benefits and Potential Toxicity

This study aimed at monitoring the levels of trace elements in table salts commercially available in markets in southern Italy and commonly used in food recipes. A balanced diet is one that provides adequate amounts of various nutrients to maintain health and well-being. The recommended dietary allowance (RDA) represents the average daily dietary intake level that is sufficient to meet the nutrient requirement of nearly all (97 to 98 per cent) healthy individuals in a group [33].

It is important to note that the elemental concentrations mentioned here are specific to the salts described and varied among different brands and production methods. Indeed, monitoring overall dietary intake and considering the cumulative contribution of elements from various food sources is essential to maintaining a balanced and healthy diet.

Focusing on the elements’ RDA for aluminum, nickel, and cobalt, there is no RDA to be followed given that these elements are not considered essential for human health. Regarding lead and mercury, being toxic substances, the preferred gourmet table salts would be those with the lowest possible content of these elements. However, chromium, iron, manganese, copper, zinc, selenium, and calcium are essential elements for the body and, therefore, the specific concentration of each element should be considered for each salt.

By assuming a daily consumption of 5 g of salt, Table 6 shows data calculated for each table salt.

The essential nutrient contents based on the recommended daily allowance (RDA) clearly indicate that certain varieties of salt, such as Atlantic grey salt, Persian blue salt, and Maldon salt, exceed the daily requirement of chromium (Cr) by over 30 mg for adults (Table 6). For adults, the recommended daily intake of calcium is approximately 800 mg although higher values are recommended for the elderly (1000 mg), adolescents, and pregnant or breastfeeding women (1200 mg) [34].

Persian blue salt contains the highest amount of calcium among the various types of salt and could potentially be beneficial for individuals with dietary deficiencies of this essential nutrient. Among the salts analyzed, Smoked salt, Guerande Grey salt, and Hawaiian pink salt, higher levels of copper were observed. The estimated daily requirement of copper for adults falls within the range of 1.5 to 3 mg [35]. However, it is worth noting that a healthy and well-balanced diet usually provides an adequate amount of copper to meet this requirement. Blue Persian salt proves to be beneficial in meeting the daily iron intake requirement, making it a recommended choice for individuals dealing with anemia [36]. Guerande salt stands out for its high manganese content while smoked salt is rich in zinc. Consequently, both types of salt can be beneficial for meeting the daily energy intake requirements, particularly for young individuals and athletes [37]. Selenium, on the other hand, had similar values across all the analyzed salts. Talking about add-on therapies, specific salts could serve as nutraceuticals in some diseases. For this reason, Persia Blue might be preferred in patients affected by osteoporosis or osteopenia. Persia Blue, Smoked, and Atlantic Grey salts might be helpful for patients with intense physical activity due to elevated levels of Zn or Guerande salt, being rich in Mn. Patients affected by anemia or a lack of iron might have beneficial effects from the intake of Persia Blue or Mozia Salt. On the contrary, Ni levels were higher in Persia Blue and Smoked salt which should not be recommended for patients affected by systemic nickel allergic syndrome (SNAS) [38]. Finally, patients with chronic kidney failure or kidney disease might consider using Smoked, Himalayan Pink, and Baule Volante which have the lowest levels of lead.

The data acquired on the minor mineral elements presented in the gourmet salts are also important for the diets of infants, toddlers, children, and adolescents. Adding these salts to their diets could lead to higher levels of deficient micronutrients but also potentially toxic and toxic elements [39,40,41]. In fact, children require higher levels of many nutrients proportional to body weight than adults and their specific needs evolve rapidly at different points in growth [39,40,41]. Due to possibly irreversible physical or cognitive deficits, the consequences of both deficiency and accumulation of mineral elements are potentially greater in children than adults [39,40,41]. Focusing on the salt samples analyzed, the intake of elements such as Ca, Fe, Mg, and Zn is potentially positive for the younger population’s health. On the contrary, the amounts of Al, Hg, Pb, and Ni added to the diet through table salts summed with the amount of Al, Hg, Pb, and Ni involuntarily present in other foods or due to contaminations might cause potential bioaccumulation phenomena which need to be monitored. Therefore, it is essential for the health and safety of the youngest in the population to know the chemical quali-quantitative minor element profiles of salts present in their dietary habits [42].

### 4.3. One Salt, One Disease

Data that have emerged highlight once more that food and natural compounds could serve as nutraceuticals. Obviously, nutraceuticals could hardly be used as drugs for treating disease but their use could help as support to traditional pharmacology or even as preventive substances in order to control and delay the development of some diseases in predisposed subjects.

Data that emerged from our analysis demonstrated that some elements are more abundant than others. Apart from Co, Cr, Cu, Hg, Pb, and Se whose levels were similar and within a tolerable range, Al, Ca, Fe, Mn, Ni, and Zn ranged significantly in the diverse salts.

Regarding add-on therapies, specific salts could serve as nutraceuticals in some diseases. For this reason, Persia Blue should be preferred in patients affected by osteoporosis or osteopenia. Persia Blue, Smoked, and Atlantic Grey salts could be useful for patients with intense physical activity due to elevated levels of Zn or Guerande salt, being rich in Mn. Patients affected by anemia or a lack of iron could benefit from the intake of Persia Blue or Mozia Salt.

On the contrary, Ni levels were higher in Persia Blue and Smoked salt which should not be recommended for patients affected by systemic nickel allergic syndrome (SNAS). Finally, patients with chronic kidney failure or kidney disease should consider using only Smoked, Himalayan Pink, and Baule Volante which have the lowest levels of lead.

## 5. Conclusions

The useful properties of different salts have been claimed for centuries. The element concentrations are fundamental for understanding the minor element profile composition of the salts, most of them unrefined. In this study, for the first time, the mineral composition of 10 salts from all over the world was investigated by ICP-MS and DMA-80 analysis to better understand their characteristics and potential benefit or harmful properties for human health. The obtained data confirmed differences among salts’ minor element chemical profiles. It warrants emphasis that despite the presence of both beneficial and potentially detrimental elements, the toxicity of these salts is not deemed a substantial concern. Standard consumption levels of these salts do not typically pose health risks. However, additional monitoring investigations on all types of table salts commercially available worldwide are necessary for human health and safety.

## Figures and Tables

**Table 1:** Salt samples list and origin area.

Samples N.	Type Codex	Type	Origin Area
3 (1a, 1b, 1c)	1	Mozia Salt	Marsala (Italy)
3 (2a, 2b, 2c)	2	Atlantic grey sea salt	-
3 (3a, 3b, 3c)	3	Persian blue salt	Iran
3 (4a, 4b, 4c)	4	Smoked salt	-
3 (5a, 5b, 5c)	5	Guerande Grey salt	Pays de la Loire (France)
3 (6a, 6b, 6c)	6	Hawaii pink salt	Hawaii islands (United States)
3 (7a, 7b, 7c)	7	Hawaii black salt	Hawaii islands (United States)
3 (8a, 8b, 8c)	8	Himalayan pink salt	Hymalayas
3 (9a, 9b, 9c)	9	Maldon salt	Maldon (United Kingdom)
3 (10a, 10b, 10c)	10	Baule Volante whole rock salt	Palermo (Italy)

**Table 2:** Analytical parameters for method validation.

Element	R^2^	LOD (µg/Kg)	LOQ (µg/Kg)	Recovery (%)
Al	0.9995	0.026	0.086	95.22 ± 2.81
Ca	0.9989	1.211	4.462	91.36 ± 2.33
Co	0.9995	0.200	0.660	94.11 ± 1.23
Cr	0.9996	0.015	0.050	96.12 ± 3.12
Cu	0.9994	0.041	0.135	96.22 ± 3.01
Fe	0.9992	0.048	0.158	93.34 ± 2.76
Hg	0.9997	0.001	0.003	98.55 ± 2.05
Mn	0.9997	0.032	0.106	95.89 ± 1.55
Ni	0.9995	0.027	0.089	98.51 ± 2.59
Pb	0.9999	0.001	0.003	101.50 ± 1.12
Se	0.9995	0.200	0.660	93.45 ± 2.43
Zn	0.9993	0.053	0.175	96.78 ± 1.89

**Table 3:** Elements’ concentration for every salt. Final values in mg/Kg were obtained considering 5 g of salt dissolved in 100 mL. The highest values are in red and the lowest values are in green.

	Mozia	Atlantic Grey	Persia Blue	Smoked	Guerande	Hawaiian Pink	Hawaiian Black	Himalayan Pink	Maldon	Baule Volante
Al	14.1	5.6	0.2	4.8	1.9	4.8	4.1	4.4	4.3	4
Ca	3438.00	2640.00	6252.00	2932.00	3818.00	2476.00	1861.40	2926.00	2624.00	2326.00
Co	0.2	0.2	0.6	0.5	0.8	0.7	0.2	0.2	0.2	0.2
Cr	0.4	0.6	1.5	0.5	0.4	0.3	0.2	0.9	0.2	0.6
Cu	41.6	35.6	50.6	32.1	30.3	30.1	28.1	28.8	29.7	28.7
Fe	10.5	2.5	21.7	1.8	1.8	1.4	6.4	2.6	2.3	2.1
Hg	0.001	0.001	0.001	0.001	0.0117	0.0016	0.001	0.0074	0.001	0.0394
Mn	2.9	1.2	3	1.1	5	2.4	2.6	1.3	0.8	1.4
Ni	0.6	0.2	12.4	7.5	0.2	1.1	1	0.4	1.5	2.2
Pb	7.2	8.4	9.2	5.2	6.6	7.9	8.2	5.5	8.4	5.6
Se	0.2	0.2	0.2	0.2	0.2	0.2	0.2	0.2	0.2	0.2
Zn	642	327.4	1334.4	340.6	178.5	215.8	156.3	246.8	238.8	215.6

**Table 4:** Average element values. Final values in mg/Kg were obtained considering 5 g of salt dissolved in 100 mL.

	Average	STDV	MAX	MIN
**Al**	4.82	3.62454595	14.1	0.2
**Ca**	3129.34	1228.16582	6252.00	1861.4
**Co**	0.38	0.24404007	0.8	0.2
**Cr**	0.56	0.3921451	1.5	0.2
**Cu**	33.56	7.26394444	50.6	28.1
**Fe**	5.31	6.42295363	21.7	1.4
**Hg**	0.00661	0.0012	0.00394	0.001
**Mn**	2.17	1.28153554	5	0.8
**Ni**	2.71	4.03414592	12.4	0.2
**Pb**	7.22	1.42111068	9.2	5.2
**Se**	0.2	0	0.2	0.2
**Zn**	389.62	359.819346	1334.4	156.3

**Table 5:** Gourmet table salt elements concentration expressed as mg/Kg. Values obtained considering 5 g of salt dissolved in 100 mL.

	Al	Ca	Co	Cr	Cu	Fe	Hg	Mn	Ni	Pb	Se	Zn
**Atlantic grey sea salt**	5.60 ± 0.21	2640.00 ± 6.28	0.21 ± 0.02	0.63 ± 0.02	35.61 ± 2.31	2.52 ± 0.32	0.001 ± 0.000	1.24 ± 0.06	0.21 ± 0.02	8.41 ± 0.33	0.21 ± 0.04	327.41 ± 3.46
**Baule** **Volante**	4.00 ± 0.24	2326.05 ± 5.88	0.21 ± 0.02	0.62 ± 0.03	28.75 ± 2.76	2.18 ± 0.30	0.04 ± 0.00	1.42 ± 0.04	2.24 ± 0.18	5.61 ± 0.18	0.21 ± 0.03	215.62 ± 1.78
**Guerande** **grey salt**	1.91 ± 0.17	3818.14 ± 4.98	0.82 ± 0.07	0.42 ± 0.02	30.36 ± 3.19	1.82 ± 0.18	0.01 ± 0.00	5.15 ± 0.62	0.23 ± 0.02	6.61 ± 0.20	0.22 ± 0.01	178.58 ± 1.45
**Hawaiian black**	4.10 ± 0.32	1861.41 ± 3.98	0.21 ± 0.02	0.22 ± 0.01	28.15 ± 2.74	6.45 ± 0.37	0.001 ± 0.000	2.64 ± 0.12	1.19 ± 0.08	8.26 ± 0.30	0.22 ± 0.02	156.38 ± 1.57
**Hawaiian** **pink**	4.81 ± 0.29	2476.06 ± 2.78	0.71 ± 0.05	0.32 ± 0.02	30.14 ± 3.55	1.44 ± 0.26	0.002 ± 0.000	2.43 ± 0.13	1.11 ± 0.06	7.92 ± 0.26	0.21 ± 0.04	215.83 ± 2.24
**Himalayan** **pink**	4.43 ± 0.51	2926.72 ± 2.65	0.20 ± 0.02	0.94 ± 0.04	28.82 ± 2.80	2.62 ± 0.28	0.01 ± 0.00	1.34 ± 0.10	0.42 ± 0.03	5.52 ± 0.27	0.20 ± 0.02	246.82 ± 2.61
**Maldon** **salt**	4.33 ± 0.22	2624.27 ± 1.99	0.21 ± 0.02	0.21 ± 0.01	29.72 ± 2.75	2.32 ± 0.28	0.001 ± 0.000	0.82 ± 0.04	1.52 ± 0.06	8.42 ± 0.32	0.20 ± 0.02	238.83 ± 2.72
**Mozia** **salt**	14.11 ± 1.03	3438.48 ± 3.77	0.21 ± 0.02	0.44 ± 0.03	41.63 ± 4.18	10.53 ± 1.27	0.001 ± 0.000	2.92 ± 0.15	0.62 ± 0.03	7.26 ± 0.25	0.21 ± 0.02	642.26 ± 5.10
**Persian** **blue salt**	0.25 ± 0.05	6252.38 ± 4.20	0.62 ± 0.03	1.53 ± 0.08	50.61 ± 6.19	21.72 ± 2.11	0.001 ± 0.000	3.17 ± 0.21	12.41 ± 1.78	9.24 ± 0.42	0.21 ± 0.01	1334.44 ± 8.17
**Smoked** **salt**	4.82 ± 0.48	2932.34 ± 2.88	0.51 ± 0.03	0.52 ± 0.02	32.13 ± 3.20	1.83 ± 0.20	0.001 ± 0.000	1.12 ± 0.10	7.52 ± 1.19	5.24 ± 0.28	0.22 ± 0.02	340.64 ± 1.90
Average	4.84 ± 0.78	3129.59 ± 2.76	0.39 ± 0.04	0.58 ± 0.07	33.59 ± 2.55	5.34 ± 1.05	0.01 ± 0.00	2.22 ± 0.22	2.75 ± 0.18	7.25 ± 0.89	0.21 ± 0.03	389.68 ± 2.76
Min.	1.91 ± 0.17	1861.41 ± 3.98	0.20 ± 0.02	0.21 ± 0.01	28.15 ± 2.74	1.44 ± 0.26	0.001 ± 0.000	0.82 ± 0.04	0.21 ± 0.02	5.24 ± 0.28	0.20 ± 0.02	156.38 ± 1.57
Max.	14.11 ± 1.03	6252.38 ± 4.20	0.82 ± 0.07	1.53 ± 0.08	50.61 ± 6.19	21.72 ± 2.11	0.04 ± 0.00	5.15 ± 0.62	12.41 ± 1.78	9.24 ± 0.42	0.22 ± 0.02	1334.44 ± 8.17

**Table 6:** Essential elements per salt with element quantities for 100 g and 5 g (maximum salt intake according FDA) and percentage of the RDA suggested by the FDA. Non-essential elements were not included.

Salts	Ca 5 g	Ca	Ca %RDA 5 g	Cr 100 g	Cr 5 g	Cr %RDA 5 g	Cu	Cu 5 g	Cu %RDA 5 g	Fe	Fe 5 g	Fe %RDA 5 g	Mn	Mn 5 g	Mn %RDA 5 g	Se	Se 5 g	Se %RDA 5 g	Zn	Zn 5 g	Zn %RDA 5 g
**Atlantic grey sea salt**	264	5280.00	20.30%	1.2	0.06	171%	71.2	3.56	396%	5	0.25	1.40%	2.4	0.12	5.20%	0.2	0.01	18.20%	654.8	32.74	298%
**Baule Volante**	232.6	4652.00	17.90%	1.2	0.06	171%	57.4	2.87	319%	4.2	0.21	1.20%	2.8	0.14	6.10%	0.2	0.01	18.20%	431.2	21.56	196%
**Guerande Grey salt**	381.8	7636.00	29.40%	0.8	0.04	114%	60.6	3.03	337%	3.6	0.18	1%	10	0.5	21.70%	0.2	0.01	18.20%	357	17.85	162%
**Hawaiian pink**	247.6	4952.00	19%	0.6	0.03	86%	60.2	3.01	334%	2.8	0.14	0.80%	4.8	0.24	10.40%	0.2	0.01	18.20%	431.6	21.58	196%
**Hawaiian black**	186.14	3722.80	14.30%	0.2	0.02	57%	56.2	2.81	312%	12.8	0.64	3.60%	5.2	0.26	11.30%	0.2	0.01	18.20%	312.6	15.63	142%
**Himalayan pink**	292.6	5852.00	22.50%	1.8	0.09	257%	57.6	2.88	320%	5.2	0.26	1.40%	2.6	0.13	5.70%	0.2	0.01	18.20%	493.6	24.68	224%
**Maldon salt**	262.4	5248.00	20.20%	0.2	0.02	57%	59.4	2.97	330%	4.6	0.23	1.30%	1.6	0.08	3.50%	0.2	0.01	18.20%	477.6	23.88	217%
**Mozia Salt**	343.8	6876.00	26.40%	0.8	0.04	114%	83.2	4.16	462%	21	1.05	6%	5.8	0.29	12.60%	0.2	0.01	18.20%	1284	64.2	584%
**Persia blue salt**	625.2	12,504.00	50.20%	3	0.15	429%	101.2	5.06	562%	43.4	2.17	12%	6	0.3	13%	0.2	0.01	18.20%	2668.8	133.44	1213%
**Smoked salt**	293.2	5864.00	22.60%	1	0.05	142%	64.2	3.21	357%	3.6	0.18	1%	2.2	0.11	4.80%	0.2	0.01	18.20%	681.2	34.06	310%
**RDA**	-	1300.00	-	0.035	-	-	0.9	-	-	18	-	-	2.3	-	-	0.055	-	-	11	-	-

## Data Availability

Not applicable.
